# A six-step approach to easy Endoloop^®^ application during laparoscopic appendicectomy

**DOI:** 10.1308/rcsann.2023.0009

**Published:** 2024-04-05

**Authors:** JQI Lim, A Dosis, M Lim

**Affiliations:** ^1^ York and Scarborough Teaching Hospitals NHS Foundation Trust, UK; ^2^ The Leeds Teaching Hospitals NHS Trust, UK

## Background

Laparoscopic appendicectomy (LA) is an index procedure for general surgical training in the United Kingdom (UK). A key step in LA involves ligation of the appendicular stump. The Endoloop^®^ (Ethicon, Bridgewater, NJ, USA) suture ligature is commonly employed and is deemed a safe and cost-effective method.^1^ However, to perform LA in a timely manner requires sufficient dexterity. We summarise our LA technique in a six-step simplified approach.

## Technique

**Figure 1:**
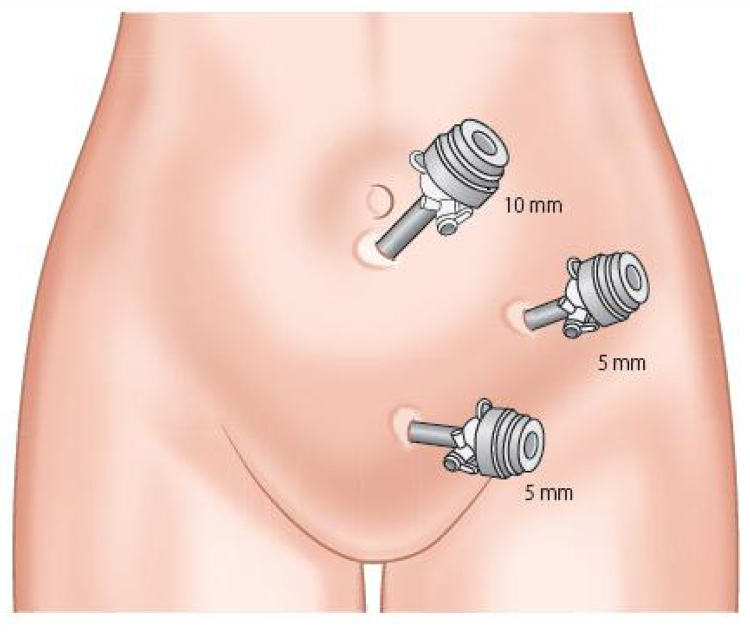
Port sizes and placement for laparoscopic appendicectomy.

Our LA technique involves three ports as illustrated in Figure 1.

After isolating the appendix from its mesentery, Endoloop^®^ is applied from the suprapubic port and the appendix retracted with a left iliac fossa grasper. The six steps are as follows:

1. insert Endoloop^®^ into patient's right iliac fossa
2. insert grasper through Endoloop^®^
3. withdraw Endoloop^®^ into port (out of camera view)
4. appendicular tip held with grasper
5. simultaneous movement of Endoloop^®^ into the right iliac fossa and grasper into the left iliac fossa port
6. simultaneous movement of Endoloop^®^ caudally and grasper cranially, focusing on traction vs counter-traction movements.

## Discussion

Endoloop^®^ application often poses a challenge to those newly introduced to the realm of laparoscopic surgery. Kim *et al* described the learning curve for LA as comprising 30 cases.^2^ By simplifying an important step of LA, training junior surgeons in cases of uncomplicated appendicitis should not contribute to additional theatre time or stress. We envisage that this technique will flatten the steep learning curve for LA and improve junior surgeons' confidence when training in a stressful theatre environment.
